# A Novel, Rapid Screening Technique for Sugar Syrup Adulteration in Honey Using Fluorescence Spectroscopy

**DOI:** 10.3390/foods11152316

**Published:** 2022-08-03

**Authors:** Sha Yan, Minghui Sun, Xuan Wang, Jihao Shan, Xiaofeng Xue

**Affiliations:** 1 College of Food Science and Engineering, Shanxi Agricultural University, Taigu 030801, China; yanshawell@163.com; 2 Institute of Apiculture Research, Chinese Academy of Agricultural Sciences, Beijing 100093, China; smh4601112@126.com (M.S.); wangxuancas@163.com (X.W.); 3 Institute of Agro-Products Processing Science and Technology, Chinese Academy of Agricultural Sciences, Beijing 100081, China; shanjihao2007@163.com

**Keywords:** honey, sugar syrup, adulteration, fluorescence spectroscopy

## Abstract

The adulteration of honey with different sugar syrups is common and difficult to detect. To ensure fair trade and protect the interests of apiarists, a rapid, simple and cost-effective detection method for adulterants in honey is needed. In this work, fluorescence emission spectra were obtained for honey and sugar syrups between 385 and 800 nm with excitation at 370 nm. We found substantial differences in the emission spectra between five types of honey and five sugar syrups and also found differences in their frequency doubled peak (FDP) intensity at 740 nm. The intensity of the FDP significantly declined (*p* < 0.01) when spiking honey with ≥10% sugar syrup. To validate this method, we tested 20 adulterant-positive honey samples and successfully identified 15 that were above the limit of detection. We propose that fluorescence spectroscopy could be broadly adopted as a cost-effective, rapid screening tool for sugar syrup adulteration of honey through characterization of emission spectra and the intensity of the FDP.

## 1. Introduction

Honey is an economically important food product worldwide. It is prized by many cultures for its pleasing aroma and flavor, and high nutritional value and potential healing properties. Given its many valuable nutritional and culinary properties, the price of natural honey is much higher than that of syrup, such as refined corn syrup (CS), rice syrup (RS), beet syrup (BS) and maltose syrup (MS). In the honey market, it is common for some merchants to attempt to take economic advantage of the differences in price by diluting honey with these less expensive sugar syrups [1,2]. Honey adulteration not only affects the quality of the honey but also harms the bee-keeping industry by negatively impacting sales and product reputation [3]. Therefore, there is an urgent need to develop effective methods for the detection of sugar syrup-adulterated honey in order to reassure consumers and ensure fair competition.

Several review papers have summarized recent studies dealing with honey adulteration, and a number of different analytical techniques are currently employed for the detection of syrup in adulterated honey [3,4,5,6]. These methodologies include common analytical techniques such as thin-layer chromatography (TLC) [7], stable carbon isotopic ratio analysis (SCIRA) [8], gas chromatography (GC) [9], high-performance anion exchange chromatography (HPAEC) [10], high-performance liquid chromatography (HPLC) [11,12] and high-performance liquid chromatography/quadrupole time of flight mass spectrometry (HPLC-Q-TOF) [3], among other emerging technologies [13,14,15]. Although these techniques offer detailed and accurate results, the processes are time-consuming and expensive and can only be performed in well-equipped laboratories by highly trained analysts.

Some advanced spectroscopic techniques have also been used for detecting honey adulteration, such as infrared spectroscopy (IR) [16,17], Raman spectroscopy (RP) [18], inductively coupled plasma optical emission spectroscopy (ICP-OES) [19] and nuclear magnetic resonance (NMR) [4,20,21], which are rapid and relatively easy but must be used in conjunction with multivariate analysis. However, the shortcoming of these methods is that they require a high level of expertise to set the chemometric models necessary to distinguish pure honey from its adulterated counterparts. Although adulteration is a global issue for the honey market, the levels and types of adulteration vary between regions. In developed nations and affluent cities, the quality of honey is generally good, and adulteration is less pervasive, since honey quality can be rigorously monitored by well-equipped testing agencies and inspectors with analytical expertise. However, in less developed regions, the regulatory infrastructure often lacks advanced analytical equipment and experts, resulting in widespread honey adulteration and often large quantities of glucose–fructose syrup [3]. There is therefore a critical need for the honey market to develop a cost-effective, simple, rapid and easy-to-use screening method for monitoring the adulteration of honey.

Fluorescence spectroscopy, a common analytical technique with relatively low-cost instrumentation that is ubiquitous in biological, food science and toxicology labs, offers several advantages for the characterization of chemical constituents, such as high sensitivity and specificity [22]. Several early studies successfully used fluorescence spectroscopy to distinguish the geographical and botanical origins of honey samples through front-face and synchronous fluorescence spectroscopy [23,24,25,26]. Fluorescence spectroscopy, in combination with chemometrics, has also been used to discriminate between true and artificial honey samples produced by feeding bee colonies with sucrose [22,27]. However, few studies have been reported on the detection of multiple adulterant sugar syrups in honey using fluorescence spectroscopy.

In the process of finding the fluorescence emission spectra of honey and different sugar syrups, we found a significant difference in the intensity of the frequency doubled peak (FDP) between honey and sugar syrup samples. The second-order diffraction of the monochromator produces the FDP. When the monochromator is set to transmit 740 nm, a small fraction of 370 nm excitation light will also be transmitted through the emission monochromator. Due to the chemical differences between syrup and honey [28], the FDP intensity at 740 nm can be influenced by different honey and sugar samples and may serve as a potential indicator to distinguish honey from sugar syrups.

We envisioned that, based on the differences in fluorescence spectra between honey and syrup, a rapid, low-cost and easy-to-use syrup-adulterated honey screening approach could be developed. In this study, we report the first application, to our knowledge, of fluorescence spectroscopy in combination with FDP intensity data for rapid screening of sugar syrup adulterants in commercial and spiked honey samples. By using the apex wavelength of characterized emission spectra in conjunction with FDP intensities as profiles, we show that natural honey from different botanical origins can be readily distinguished from honey adulterated with a range of different syrups without the need for multivariate analysis. Our findings represent a critical and clear step toward the application of common fluorescence spectroscopy in the screening for honey adulteration.

## 2. Materials and Methods

### 2.1. Sample Collection

To examine the fluorescence emission spectra and FDP of pure honey from different botanical origins, we sampled five common types of commercial honey produced across China and derived from *Robinia pseudoacacia* L. (acacia), *Brassica napus* L. (rape), *Tilia tuan* *Szyszy* L. (medlar), *Lycium barbarum* L. (linden) and *Vitex negundo* L. (chaste). A total of 112 honey samples, including acacia honey (32, labeled A1–A32), rape honey (32, labeled R1–R32), medlar honey (18, labeled J1–J18), linden honey (15, labeled L1–L15) and chaste honey (15, labeled V1–V15), were obtained from twenty-five collaborating beekeepers located in Zhejiang Province, Sichuan Province, Shandong Province, Shaanxi Province, Jilin Province, Xinjiang Province and Beijing. The entirety of the collection process, for all honey samples, was monitored and recorded to ensure sample authenticity. All honey samples were collected from capped combs, ensuring all samples were ripe and mature. The moisture content of the samples was ≤19.0%.

Thirty-one sugar syrup samples, produced by different companies, were purchased from different markets located in Sichuan, Henan, Shandong and Jiangsu Provinces. These samples included ten high-fructose corn syrups (HFCS1−HFCS10), six rice syrups (RS1−RS6), five beet syrups (BS1−BS5), five cassava syrups (CS1−CS5) and five maltose syrups (MS1−MS5).

Twenty adulterant-positive acacia honey samples (sam1–sam20) were graciously provided by an independent testing lab of the Shandong Bee Industry and Bee Products Quality Monitoring. The glucose, fructose, sucrose and maltose contents were determined by the high-performance liquid chromatography-refractive index detection (HPLC-RID) method, as described in [3,5]. According to [29], the stable carbon isotopic ratio analysis was performed using an isotope ratio mass spectrometer (SerCon EA, Wistaston, UK) to determine ^13^C/^12^C ratios. The TLC test was performed following the procedures in [7,10].

### 2.2. Sample Preparation

HFCS, RS, BS, CS and MS were mixed and added to pure honey samples in a range of concentrations (10%, 20%, 30%, 90%, *w*/*w*) for analysis.

For sample pretreatment, 10 g of honey sample and 10 mL of deionized water were added to a 50 mL centrifuge tube and mixed by vortex for 3 min. The resulting sample solutions were directly used for subsequent fluorescence spectroscopy analysis.

### 2.3. Fluorescence and Frequency Doubled Peak (FDP) Measurements

The fluorescence measurements were conducted with three brands of fluorescence spectrophotometers including a Hitachi F-4500 (Tokyo, Japan), a Shimadzu RF-5301PC (Shimadzu, Japan) and a Shanghai Lingguang F97 (Shanghai, China), which were all equipped with a plotter unit and a 1 cm quartz cell. The fluorescence of each sample solution was directly measured with an excitation wavelength of 370 nm and an emission range from 385 to 800 nm. The scanning speed was 12,000 nm/min, and spectrometer slits were set for a 5 nm band-pass. Fluorescence measurements of each sample were carried out in triplicate. The pattern of fluorescence spectra and the FDP intensity of all samples were recorded at room temperature.

### 2.4. Statistical Analyses

The results were expressed using the means ± standard deviations. SPSS 19.0 (IBM, Stamford, CT, USA) was used to analyze significant differences, and ANOVA (LSD test) was performed to determine significant differences.

## 3. Results and Discussion

### 3.1. Fluorescence Spectra Characteristics of Honey and Syrup

**Figure 1:**
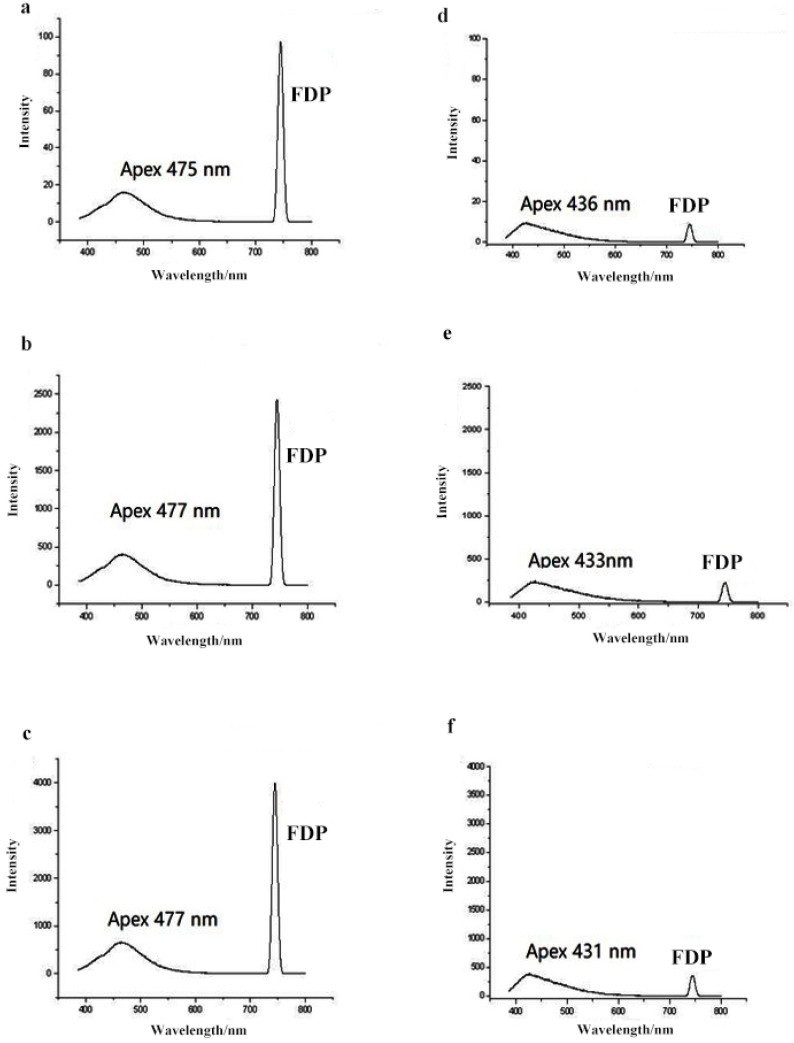
Comparison of honey and syrup fluorescence spectra generated by fluorescence spectrophotometers from three different manufacturers ((**a**) pure honey analyzed by a Hitachi F-4500; (**b**) pure honey analyzed by a Lingguang F97; (**c**) pure honey analyzed by a Shimadzu RF-5301PC; (**d**) syrup analyzed by a Hitachi F-4500; (**e**) syrup analyzed by a Lingguang F97; (**f**) syrup analyzed by a Shimadzu RF-5301PC).

To first determine if the FDP is a common phenomenon among fluorescence spectrophotometers from different manufacturers, and to test for variation between instruments, the fluorescence spectra for each honey type and sugar syrup were measured by the three brands of fluorescence spectrophotometers (Figure 1). By removing the specific optical filter during the acquisition process, the results show that the FDP is a common phenomenon among the different manufacturers. Furthermore, the application of fluorescence spectroscopy in combination with measurements of the FDP intensity can be used as a suitable indicator for the adulteration of honeys with a variety of sugar syrups.

Although we found differences in the absolute, or quantitative, measurements between instruments from the different manufacturers, the apex and the area ratio of fluorescence emission spectra and the area ratio of the FDP between honey and syrup were uniform across all three instruments (Table S2). Data in our study were collected using a Hitachi F-4500 (Tokyo, Japan).

### 3.2. The Fluorescence Spectra Profiles of Different Honeys and Syrups

**Figure 2:**
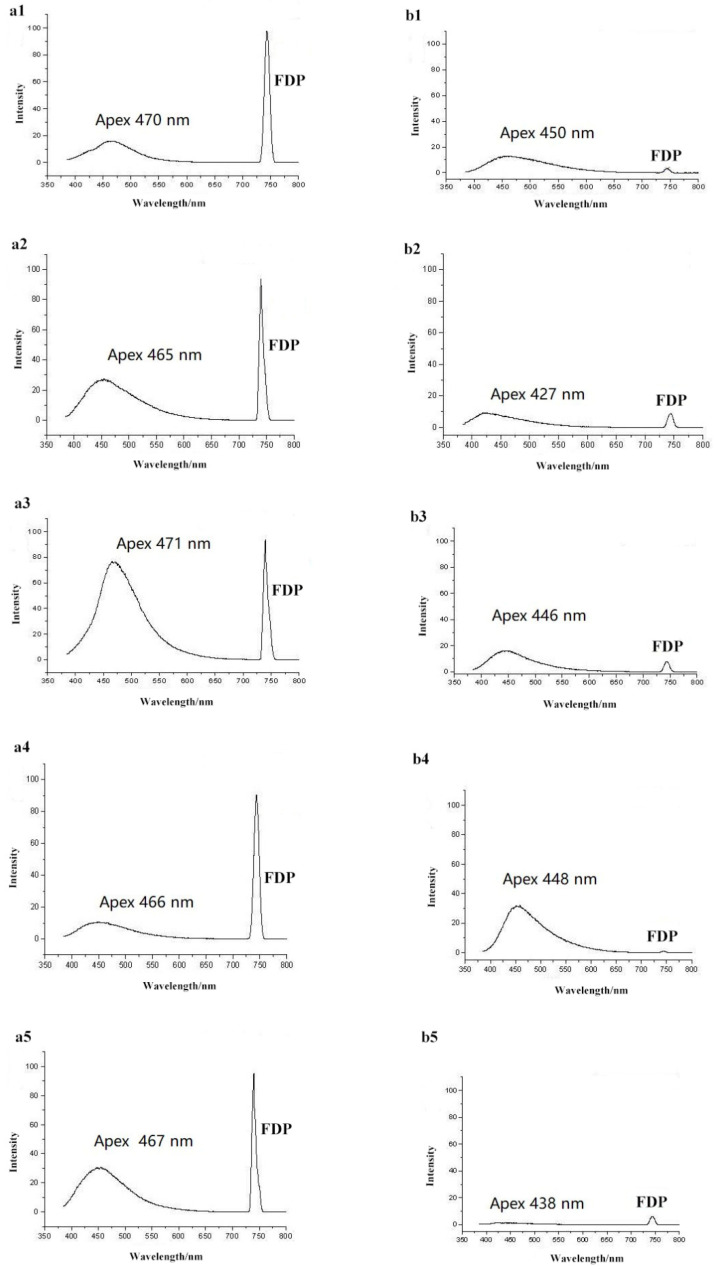
Typical fluorescence spectra of honey and syrup ((**a1**): acacia honey; (**a2**): chaste honey; (**a3**): medlar honey; (**a4**): rape honey; (**a5**): linden honey; (**b1**): maltose syrup; (**b2**): HFCS; (**b3**): rice syrup; (**b4**): beet syrup; (**b5**): cassava syrup).

To aid in the characterization of honey and syrup profile spectra, we acquired published information on fluorescence spectra from previous research on honey and syrup samples [22,23,24,25,26]. Compositionally, honey consists of sugars and water with small amounts of proteins, free amino acids, peptides, minerals, vitamins, organic acids, flavonoids and other phenolic compounds and aroma compounds. These constituents, and especially many of the minority components, exhibit fluorescent properties [30]. For example, a peak with an excitation of 280 nm and emission of 340 nm can potentially indicate fluorescence from aromatic amino acids present in the honey [22]. A fluorescence peak with an emission of 450 nm and excitation at 250 nm can be attributed to non-flavonoid phenolic compounds in the honey sample [31]. The Maillard reaction products, such as hydroxymethylfurfural and furosine, can have characteristic fluorescence peaks with emission wavelengths ranging from 420 to 470 nm and excitation wavelengths between 340 and 380 nm [22]. We compared the fluorescence emission spectra excitation at 250~380 nm. The results show that all honeys had a fixed apex wavelength at 468 nm, and the apex wavelengths of all syrups were 442 nm. Additionally, the intensities of the FDP showed a visible difference between the honey and syrup samples. Therefore, in this study, fluorescence spectra for each pure honey and pure sugar syrup were examined by excitation at 370 nm and data acquisition from 385 to 800 nm (Figure 2). The fluorescence emission spectra were clearly observable in the 385 nm to 700 nm range, with the FDP appearing at 740 nm. Spectral profiles were visibly different between honey and syrup samples, especially in the presence and size of the FDP.

In comparison to natural honey, the fluorescence emission spectra of the syrups produced a blueshift of about 26 nm. Detailed values of the five syrup types and honeys are shown in Table 1. The apexes of the fluorescence emission spectra from all five honeys were located at 470 nm, while the apexes of the syrups were centered at 450 nm. This shift is potentially attributable to differences in the array of conjugated compounds such as polyphenols, aromatic amino acids, Maillard reaction products and other small molecules present in the samples. Ghosh and colleagues investigated the fluorescence spectroscopic properties of honey and cane sugar syrup and found that all spectra from pure honey samples were characterized by two prominent features: a shoulder around 440 nm and a broad band around 510 nm. In contrast, a single prominent band around 430 nm was characteristic of spectra from cane sugar syrup. In addition, their analysis revealed that the major contributor to cane sugar syrup fluorescence is the reduced form of nicotinamide adenine dinucleotide, while the fluorescence of honey is predominantly caused by flavins [32].

The areas of the emission spectra were substantially different between the different types of honey, possibly due to plant sources, geographic origins and climatic factors, all of which can influence the content of conjugated compounds. The same feature is true for the syrup spectral data. Furthermore, FDPs were highest at 740 nm for pure honey samples but exhibited a very low intensity in the syrup samples (values listed in Table 1). The FDP area for pure honey was consistently 10-fold greater than that of pure syrup. We propose that this difference between the FDP intensity of pure honey and that of syrup could be exploited to monitor adulteration by all types of syrups.

### 3.3. The Fluorescence Spectra Profiles of Adulterated Honey

Previous studies have examined fluorescence spectra as a means of distinguishing between pure honey and sugar syrup as well as between honeys from different botanical and geographical origins [23]. In light of published articles describing the features of emission spectra, we found that the incorporation of the FDP intensity with these known spectra allowed us to develop profiles for the identification of sugar syrup adulterants in honey.

We blended beet syrup, cassava syrup, malt syrup, rice syrup and HFCS and incrementally added the mixture (0, 10%, 20%, 30%…90%) to a blend of all five honeys to assess the correlation between the amount of added syrup and FDP intensities. The apex wavelengths and areas of the fluorescence emission spectra, as well as FDP intensities (Table 2), revealed that as the proportion of syrup increased, a significant blueshift (with the honey peak at 468 nm and the syrup peak at 442 nm) occurred, and the FDP areas decreased in a dose-dependent gradient. Thus, the FPD was positioned at 740 nm, and measurement of its intensity combined with the apex wavelength of the fluorescence emission spectra could be readily used to discriminate between pure and adulterated honey samples. As Figure S1 shows, the *R*^2^ between the amounts of adulterated syrup and FDP intensities was 0.9873 (*p* < 0.01). Thus, the FDP intensity can strongly reflect the amount of syrup added.

**Figure 3:**
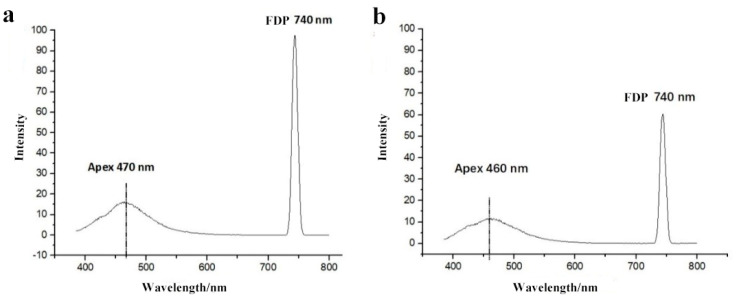
Typical fluorescence spectra of adulterated acacia honey with 30% syrup ((**a**): pure honey; (**b**): acacia honey adulterated with 30% syrup).

Commonly, the addition of 30% or higher syrup can make substantial profits. In this study, the minimum amount of syrup added was 10%, and compared with the honey sample, there was a significant difference (*p* < 0.01) (Table 2). We also found that a small deviation in the apex wavelength could be used as an auxiliary qualitative indicator for the relative purity of a given honey sample. Specifically, the apex wavelength shifted from 470 nm to 460 nm, and the FDP intensity decreased from 990 to 610 with the dilution of acacia honey to 30% syrup (Figure 3).

To verify the accuracy of the method, we chose acacia honey samples from different sources and different collection times to compare the intensities of the FDP. Among these acacia honey samples, there were no significant differences (*p* > 0.05) (for detailed information, see Table S1). Thus, the samples with the addition of at least 10% syrup could be identified by significance analysis (*p* < 0.01) compared with natural honey samples.

### 3.4. Application to Adulterated Market Samples

Acacia honey, from blossoms of *Robinia pseudoacacia*, is a transparent, pale-yellow honey, valued for its mild flavor compared to other honey varieties. In general, the yield of acacia honey is lower than for other honeys, and thus its market price is higher. Syrup adulteration of acacia honey is currently a serious problem [33,34]. Therefore, adulterant-positive samples of acacia honey were used to verify the effectiveness of our method (Table 3). Twenty samples were taken from local, small markets in Shandong and Henan.

As part of this study, we compared a method for glucose, fructose, sucrose and maltose analysis to effectively identify maltose syrup [5]. Similarly, we examined stable carbon isotope ratio analysis [29] for the identification of C4 plant sugar (corn syrup) in adulterated honey and found that, although it is effective with high accuracy for HFCS, C3 plant sugars (e.g., beet, rice and maltose syrup) cannot be readily distinguished in samples due to the similarity of their carbon isotope content to that of natural honey [3]. A third method, TLC [7,10], has also been used to target unhydrolyzed polysaccharides and oligosaccharides in some corn and maltose syrups. Each of these methods has unique advantages, but none of them is uniformly effective for the identification of all syrup types in adulterated honey.

The fluorescence-based method demonstrated in our study is a simple and relatively fast screening method to identify high-fructose corn syrup as well as beet, rice and maltose syrups that have been added to honey, with a reasonably low limit of detection at 10%. We found that five adulterated honey samples, one with rice syrup and four with HFCS, could not be identified (false negatives) because of the low syrup content, and thus, due to the detection limit, we conclude that the accuracy of the method is 75%. Among the fifteen positive samples, the apex wavelength ranged from 441 to 452 nm, and FDP intensities were below 200 (compared to the fluorescence spectra information of the syrups, see Table 1). These results indicate that these positive samples are almost pure syrup. In order to maximize profit, in some regions, samples can be found that are composed of almost 100% pure syrup. In these places, where effective market regulation is needed most, our method provides a fast and practical approach for the detection of adulterated honey with a high syrup content.

### 3.5. Interpretation of Differences in FDP between Honey and Syrup

Under 370 nm excitation, there was an FDP at 740 nm that could be used to distinguish between honey and syrup samples. The FDP arises in non-linear materials. When the monochromator was set to transmit 740 nm, a small fraction of 370 nm excitation light was also transmitted through the emission monochromator. Thus, the observed differences in the FDP at 740 nm were due to the differences in the 370 nm transmission through the samples. The difference in the FDP intensity between honey and syrup may be caused by differences in the amounts and types of macromolecular polymers in each sweetener, such as proteins or nucleotides. Different sources and processing techniques of honey and syrup are also likely to produce differences in the types and polymerization states of macromolecules, thus resulting in measurably large variation in the FDP between products. Interestingly, we found that jujube and buckwheat honeys are not clearly distinguishable from syrup using this method, due to their low FDP intensity, and we thus speculate that this method is currently unsuitable for dark honey. However, the underlying reasons and mechanisms require further study, and with some optimization, this technique may be modified for broader use.

## 4. Conclusions

We reported the development of a novel fluorescence screening method combining the FDP intensity with the apex wavelength of fluorescence emission spectra in order to identify syrup adulterants in honey. We demonstrated that the FDP intensity decreases significantly and the apex wavelength undergoes a distinct, dose-dependent blueshift with the addition of increasing amounts of syrup to pure honey. Using this method, 10% syrup and higher can be detected in adulterated honey. To validate this method, we analyzed 20 syrup-adulterated honey samples and successfully detected 11 out of 15 with HFCS, 1 out of 2 with rice syrup, 2 out of 2 with beet syrup and 1 (out of 1) with maltose syrup, thus demonstrating 75% accuracy. In comparison with other more sophisticated, labor-intensive and expensive spectroscopy methods, the analysis time was less than 1 min, and the instrument can be operated with minimal training and no subsequent statistical analyses to interpret the data. For most consumers, honey is purchased for its nutritional and medicinal benefits, and especially for its purity. This is also the first report, to our knowledge, on the application of the FDP to research on food adulteration. These experiments provide an exciting route toward wide adoption of this technique by honey producers and regulatory agencies.

## Figures and Tables

**Table 1:** Apex wavelength and area of emission spectra and FDP area of honeys and syrups.

Honey	Fluorescence Emission Spectra		FDP
	Apex Wavelength (nm)	Area ± RSD	Area ± RSD
Medlar honey	471 ± 2 nm	3783 ± 169.3	1088 ± 30.4
Acacia honey	470 ± 3 nm	1897 ± 50.7	1221 ± 29.7
Linden honey	467 ± 4 nm	3324 ± 151.5	1006 ± 20.4
Chaste honey	465 ± 3 nm	3151 ± 80.1	935 ± 19.9
Rape honey	466 ± 2 nm	1615 ± 26.2	1080 ± 29.6
Average	468 ± 2.8 nm	2754 ±95.6	1064 ± 26.0
Syrup			
Beet syrup	448 ± 4 nm	3280 ± 189.2	34 ± 5.1
Cassava syrup	438 ± 3 nm	573 ± 56.5	181 ± 16.8
Malt syrup	450 ± 3 nm	1664 ± 21.1	46 ± 4.9
Rice syrup	445 ± 2 nm	619 ± 30.8	103 ± 9.6
HFCS	427 ±5 nm	2311 ± 230.0	53 ± 5.5
Average	442 ± 3.4 nm	1689 ± 105.2	83.4 ± 8.4

**Table 2:** Fluorescence spectra information of artificially adulterated honey.

Items	Fluorescence Emission Spectra		FDP
	Apex (nm)	Area ± RSD	Area ± RSD
Honey	472 ± 5 nm	1699 ± 41.9	976 ± 35.8 a
10% syrup	468 ± 4 nm	2605 ± 42.8	813 ± 39.0 b
20% syrup	467 ± 5 nm	2530 ± 41.6	765 ± 33.2 bc
30% syrup	461 ± 4 nm	2375 ±39.3	713 ± 32.0 cd
40% syrup	460 ± 5 nm	2270 ± 38.3	623 ± 34.7 de
50% syrup	458 ± 4 nm	2076 ± 37.6	584 ± 28.9 ef
60% syrup	458 ± 6 nm	1873 ± 36.4	503 ± 25.6 fg
70% syrup	454 ± 5 nm	1776 ± 30.9	410 ± 27.5 gh
80% syrup	449 ± 3 nm	1519 ± 27.1	335 ± 25.5 h
90% syrup	446 ± 2 nm	1267 ± 21.6	200 ± 30.4 i

Note: lowercase letters indicate a significant difference (*p* < 0.01).

**Table 3:** Results from four different methods.

Positive Sample Information	Total Numbers	Glucose, Fructose, Sucrose and Maltose Analysis	Stable Carbon Isotopic Ratio Analysis	TLC Method	Fluorescence Method
High-fructose corn syrup-adulterated honey	15	+(0) ^a^	+(15)	+(4)	+(11)
Beet syrup-adulterated honey	2	+(0)	+(0)	+(0)	+(2)
Rice syrup-adulterated honey	2	+(0)	+(0)	+(0)	+(1)
Maltose syrup-adulterated honey	1	+(1)	+(0)	+(1)	+(1)

^a^ Adulterated samples are indicated with +; parentheses indicate the number of samples.

## Data Availability

Data are contained within the article.

## Supplementary Materials

The following supporting information can be downloaded at: https://www.mdpi.com/article/10.3390/foods11152316/s1, Figure S1: The relationship of amount of syrup added and area of FDP. Table S1: Fluorescence Spectra Information of acacia honey Samples. Table S2: The comparison of fluorescence spectra information from 3 different manufacturers.

## References

[1] Osman K.A., Al-Doghairi M.A., Al-Rehiayani S., Helal M.I.D. (2007). Mineral contents and physicochemical properties of natural honey produced in Al-Qassim region, Saudi Arabia. J. Food Agric. Environ..

[2] Al M.L., Daniel D., Moise A., Bobis O., Laslo L., Bogdanov S. (2009). Physico-chemical and bioactive properties of different floral origin honeys from Romania. Food Chem..

[3] Wu L., Du B., Vander Heyden Y., Chen L., Zhao L., Wang M., Xue X. (2017). Recent advancements in detecting sugar-based adulterants in honey—A challenge. TrAC Trends Anal. Chem..

[4] Siddiqui A.J., Musharraf S.G., Choudhary M.I. (2017). Application of analytical methods in authentication and adulteration of honey. Food Chem..

[5] Naila A., Flint S.H., Sulaiman A.Z., Ajit A., Weeds Z. (2018). Classical and novel approaches to the analysis of honey and detection of adulterants. Food Control.

[6] Tsagkaris A.S., Koulis G.A., Danezis G.P., Martakos I., Dasenaki M., Georgiou C.A., Thomaidis N.S. (2021). Honey authenticity: Analytical techniques, state of the art and challenges. RSC Adv..

[7] Puscas A., Hosu A., Cimpoiu C. (2013). Application of a newly developed and validated high-performance thin-layer chromatographic method to control honey adulteration. J. Chromatogr. A.

[8] Guler A., Kocaokutgen H., Garipoglu A.V., Onder H., Ekinci D., Biyik S. (2014). Detection of adulterated honey produced by honeybee (*Apis mellifera* L.) colonies fed with different levels of commercial industrial sugar (C3 and C4 plants) syrups by the carbon isotope ratio analysis. Food Chem..

[9] Ruiz-Matute A.I., Soria A.C., Martínez-Castro I., Sanz M.L. (2007). A new methodology based on GC-MS to detect honey adulteration with commercial syrups. J. Agric. Food Chem..

[10] Megherbi M., Herbreteau B., Faure R., Salvador A. (2009). Polysaccharides as a Marker for Detection of Corn Sugar Syrup Addition in Honey. J. Agric. Food Chem..

[11] Xue X., Wang Q., Li Y., Wu L., Chen L., Zhao J., Liu F. (2013). 2-acetylfuran-3-glucopyranoside as a novel marker for the detection of honey adulterated with rice syrup. J. Agric. Food Chem..

[12] Wang S., Guo Q., Wang L., Lin L., Shi H., Cao H., Cao B. (2015). Detection of honey adulteration with starch syrup by high performance liquid chromatography. Food Chem..

[13] Sobrino-Gregorio L., Bataller R., Soto J., Escriche I. (2018). Monitoring honey adulteration with sugar syrups using an automatic pulse voltammetric electronic tongue. Food Control.

[14] Sobrino-Gregorio L., Vilanova S., Prohens J., Escriche I. (2019). Detection of honey adulteration by conventional and real-time PCR. Food Control.

[15] de Souza R.R., Fernandes D.D.S., Diniz P. (2021). Honey authentication in terms of its adulteration with sugar syrups using UV-Vis spectroscopy and one-class classifiers. Food Chem..

[16] Kelly J.D., Petisco C., Downey G. (2006). Application of Fourier Transform Midinfrared Spectroscopy to the Discrimination between Irish Artisanal Honey and Such Honey Adulterated with Various Sugar Syrups. J. Agric. Food Chem..

[17] Chen L., Xue X., Ye Z., Zhou J., Chen F., Zhao J. (2011). Determination of Chinese honey adulterated with high fructose corn syrup by near infrared spectroscopy. Food Chem..

[18] Li S., Yang S., Zhu X., Zhang X., Ling G. (2012). Detection of honey adulteration by high fructose corn syrup and maltose syrup using Raman spectroscopy. J. Food Compos. Anal..

[19] Liu T., Ming K., Wang W., Qiao N., Qiu S., Yi S., Huang X., Luo L. (2021). Discrimination of honey and syrup-based adulteration by mineral element chemometrics profiling. Food Chem..

[20] Bertelli D., Lolli M., Papotti G., Bortolotti L., Serra G., Plessi M. (2010). Detection of Honey Adulteration by Sugar Syrups Using One-Dimensional and Two-Dimensional High-Resolution Nuclear Magnetic Resonance. J. Agric. Food Chem..

[21] Guelpa A., Marini F., du Plessis A., Slabbert R., Manley M. (2017). Verification of authenticity and fraud detection in South African honey using NIR spectroscopy. Food Control.

[22] Lenhardt L., Bro R., Zeković I., Dramićanin T., Dramićanin M.D. (2015). Fluorescence spectroscopy coupled with PARAFAC and PLS DA for characterization and classification of honey. Food Chem..

[23] Ruoff K., Karoui R., Dufour E., Luginbühl W., Bosset J.O., Bogdanov S., Amado R. (2005). Authentication of the Botanical Origin of Honey by Front-Face Fluorescence Spectroscopy. A Preliminary Study. J. Agric. Food Chem..

[24] Ruoff K., Luginbühl W., Künzli R., Bogdanov S., Bosset J.O., von der Ohe K., von der Ohe W., Amadò R. (2006). Authentication of the Botanical and Geographical Origin of Honey by Front-Face Fluorescence Spectroscopy. J. Agric. Food Chem..

[25] Sergiel I., Pohl P., Biesaga M., Mironczyk A. (2014). Suitability of three-dimensional synchronous fluorescence spectroscopy for fingerprint analysis of honey samples with reference to their phenolic profiles. Food Chem..

[26] Mehretie S., Al Riza D.F., Yoshito S., Kondo N. (2018). Classification of raw Ethiopian honeys using front face fluorescence spectra with multivariate analysis. Food Control.

[27] Santana J.E.G., Coutinho H.D.M., da Costa J.G.M., Menezes J.M.C., Pereira Teixeira R.N. (2021). Fluorescent characteristics of bee honey constituents: A brief review. Food Chem..

[28] Valinger D., Longin L., Grbeš F., Benković M., Jurina T., Gajdoš Kljusurić J., Tušek A.J. (2021). Detection of honey adulteration—The potential of UV-VIS and NIR spectroscopy coupled with multivariate analysis. LWT-Food Sci. Technol..

[29] Cabañero A.I., Recio J.L., Rupérez M. (2006). Liquid chromatography coupled to isotope ratio mass spectrometry: A new perspective on honey adulteration detection. J. Agric. Food Chem..

[30] Hao S., Li J., Liu X., Yuan J., Yuan W., Tian Y., Xuan H. (2021). Authentication of acacia honey using fluorescence spectroscopy. Food Control.

[31] Kečkeš S., Gašić U., Veličković T.Ć., Milojković-Opsenica D., Natić M., Tešić Ž. (2013). The determination of phenolic profiles of Serbian unifloral honeys using ultra-high-performance liquid chromatography/high resolution accurate mass spectrometry. Food Chem..

[32] Ghosh N., Verma Y., Majumder S.K., Gupta P.K. (2005). A fluorescence spectroscopic study of honey and cane sugar syrup. Food Sci. Technol. Res..

[33] Kenjerić D., Mandić M.L., Primorac L., Bubalo D., Perl A. (2007). Flavonoid profile of Robinia honeys produced in Croatia. Food Chem..

[34] Wang J., Xue X., Du X., Cheng N., Chen L., Zhao J., Zheng J., Cao W. (2014). Identification of acacia honey adulteration with rape honey using liquid chromatography–electrochemical detection and chemometrics. Food Anal. Method.

