# Supplementary material for: A Novel, Rapid Screening Technique for Sugar Syrup Adulteration in Honey Using Fluorescence Spectroscopy
Source: Foods. 2022 Aug 3;11(15):2316. doi: 10.3390/foods11152316 (PMC9368237; doi:10.3390/foods11152316)
Supplement: Supplementary file 1 [file foods-11-02316-s001.zip › foods-1822026-supplementary.pdf]

A Novel, Rapid Screening Technique for Sugar Syrup Adulteration in Honey using Fluorescence Spectroscopy

Sha Yan[1, 2]**, Minghui Sun**[2]**, Xuan Wang**[2]**, Jihao Shan**[3]**, Xiaofeng Xue**[b, *]

[1] *College of Food Science and Engineering, Shanxi Agricultural University, Taigu 030801, China*

[2] *Institute of Apiculture Research, Chinese Academy of Agricultural Sciences, Beijing 100093, China*

[3] *Institute of Agro-Products Processing Science and Technology, Chinese Academy of Agricultural Sciences，Beijing 100081, China*

* Corresponding author. Tel./fax: +86 10 62594643

E-mail addresses: xue_xiaofeng@126.com (X. F. Xue)

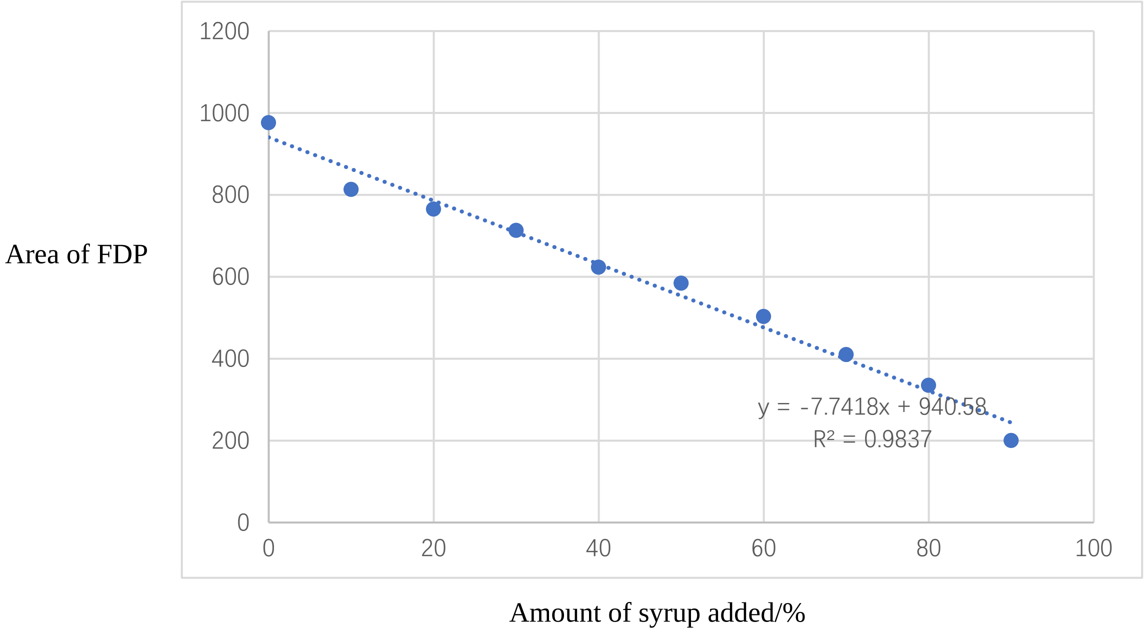

Figure S1. The relationship of amount of syrup added and area of FDP

Table S1. Fluorescence Spectra Information of acacia honey samples

Source	Collection time	Fluorescence emission spectra		FDP
		Apex (nm)	Area ± RSD	Area ± RSD
Beijing	2019.06	470 ± 5	1899 ± 43.3	1086 ± 33.6 a
	2020.05	470 ± 3	1876 ± 30.2	1104 ± 25.8 a
	2020.06	470 ± 5	1920± 41.9	1072 ± 30.9 a
Shannxi	2019.06	470 ± 3	1893 ±36.3	1181 ± 32.0 a
	2020.06	470 ± 5	1852 ± 48.1	1207 ± 16.7 a
	2021.06	470 ± 4	1901 ± 31.5	1226 ± 39.3 a
Shandong	2019.06	470 ± 5	1871 ± 50.6	1291 ± 40.1 a
	2020.06	470 ± 5	1876 ± 20.9	1208 ± 26.4 a
	2021.06	470 ± 6	1921 ± 53.6	1200 ± 38.8 a

Note: Lowercase letters indicate significant difference ($p < 0.05$)

Table S2 The comparison of fluorescence spectra information from 3 different manufacturers

Manufacturer	Fluorescence emission spectra		FDP
	Apex (nm)	Area ratio (Acacia honey/Beet syrup)	Area ratio (Acacia honey/Beet syrup)
Hitachi F-4500	470 ± 3nm (Acacia honey)<br>448 ± 4nm (Beet syrup)	0.58	32
Shimadzu RF-5301PC	470 ± 3nm (Acacia honey)<br>448 ± 4nm (Beet syrup)	0.60	32
Lingguang F97	470 ± 3nm (Acacia honey)<br>448 ± 4nm (Beet syrup)	0.58	31.5
